# Sequence and gene expression of chloroquine resistance transporter (*pfcrt*) in the association of *in vitro *drugs resistance of *Plasmodium falciparum*

**DOI:** 10.1186/1475-2875-10-42

**Published:** 2011-02-15

**Authors:** Wanna Chaijaroenkul, Stephen A Ward, Mathirut Mungthin, David Johnson, Andrew Owen, Patrick G Bray, Kesara Na-Bangchang

**Affiliations:** 1 Faculty of Allied Health Sciences, Thammasat University, Rangsit, Patumthani 12120, Thailand; 2 Division of Molecular and Biochemical Parasitology of Liverpool, University of Liverpool, Pembroke Place, Liverpool L35QA, UK; 3 Department of Parasitology, Phramongkutklao College of Medicine, Ratchathewi, Bangkok 10400, Thailand; 4 Department of Pharmacology and Therapeutics, University of Liverpool, Pembroke Place, Liverpool L35QA, UK

## Abstract

**Background:**

*Plasmodium falciparum *chloroquine resistance (CQR) transporter protein (PfCRT) is known to be the important key of CQR. Recent studies have definitively demonstrated a link between mutations in the gene *pfcrt *and resistance to chloroquine in *P. falciparum*. Although these mutations are predictive of chloroquine resistance, they are not quantitatively predictive of the degree of resistance.

**Methods:**

In this study, a total of 95 recently adapted *P. falciparum *isolates from Thailand were included in the analysis. Parasites were characterized for their drug susceptibility phenotypes and genotypes with respect to *pfcrt*. From the original 95 isolates, 20 were selected for complete *pfcrt *sequence analysis.

**Results:**

Almost all of the parasites characterized carried the previously reported mutations K76T, A220S, Q271E, N326S, I356T and R371I. On complete sequencing, isolates were identified with novel mutations at K76A and E198K. There was a suggestion that parasites carrying E198K were less resistant than those that did not. In addition, *pfcrt *and *pfmdr1 *gene expression were investigated by real-time PCR. No relationship between the expression level of either of these genes and response to drug was observed.

**Conclusion:**

Data from the present study suggest that other genes must contribute to the degree of resistance once the resistance phenotype is established through mutations in *pfcrt*.

## Background

Malaria, especially that caused by *Plasmodium falciparum*, is a serious health problem in many tropical countries and this situation is made worse as a consequence of drug resistance. The 4-aminoquinoline chloroquine (CQ) was introduced in the 1940's as a cheap and effective treatment for malaria. Chloroquine-resistant (CQR) parasites were first reported in the late 1950s from South-East Asia and South-America. Since those early reports, CQR *P. falciparum *has spread throughout the malaria endemic world. In addition, in some areas such as the Thai-Myanmar and the Thai-Cambodian borders, parasite populations resistant to most available drugs have evolved. These parasites have been referred to as multidrug-resistant (MDR) *P. falciparum *[1].

Reduced dug accumulation is a phenotypic feature of CQR which can be partially reversed by the calcium channel blocker verapamil, an agent that also reverses chloroquine resistance [2-5]. Two genes have been linked with this phenotype namely *pfmdr1 *and *pfcrt*. The *pfmdr1 *gene is located on chromosome 5 and *pfcrt *gene is located on chromosome 7. The weight of molecular evidence suggests that while *pfmdr1 *may have a modulatory effect in parasite susceptibility to CQ [6], mutation in *pfcrt *is the principal determinant of CQR [7].

Several mutations have been identified in the *pfcrt *gene. CQR *P. falciparum *strains from South East Asia and Africa carry point mutations at codons 74, 75, 76, 220, 271, 326 and 371, whereas, CQR South American parasites carry point mutations at codons 76, 220 and either 72, 326 and 356 or 75, 97 and 371 [8]. The (K76T) mutation is strongly associated with CQ susceptibility and clinical outcomes. Transfection studies have confirmed the importance of this amino acid change [9]. The PfCRT protein is localized to the digestive vacuole membrane and appears to be an integral membrane protein with an as yet unidentified transport function. Hypotheses have been proposed to explain CQR that implicate PfCRT function as either an ion channel or as a direct drug transporter. In addition to mutation in *pfcrt *being involved in CQR, it has also been suggested that the level of *pfcrt *expression has an impact on parasite susceptibility [10]. Waller *et al *demonstrated that lines transfected with *pfcrt *alleles associated with CQR displayed a 30-40% reduction in protein expression together with an increase in CQ sensitivity [10]. In contrast, Durrand *et al *failed to identify any correlation between the CQ response of field isolates and *pfcrt *gene expression [11].

It is generally accepted that PfCRT is the principal determinant of CQR. However, it is not possible to predict the degree of CQR based on the *pfcrt *genotype alone or even in combination with *pfmdr1 *genotype. It is clear form our own surveillance studies and those of others that parasites considered to be CQR in actual fact display a broad range of sensitivity to the drug. In this study, the phenotype-genotype relationship between 95 recently adapted isolates of *P. falciparum *from Thailand were characterized. Furthermore, 20 selected isolates were fully sequenced for *pfcrt *gene in order to look for novel mutations that might be implicated in the degree of CQR.

## Methods

### Parasite isolates

Clinical *P. falciparum *isolates were collected from malaria endemic areas of Thailand including the Thai-Myanmar (Kanchanaburi, Tak, Ratchaburi and Ranong Provinces) and Thai-Combodian borders (Chantaburi Province) between 1998 and 2003 prior to the initiation of antimalarial treatment. Blood samples from individual patients were collected under a clinical protocol approved by the Ethics Committee of the Ministry of Public Health of Thailand. Briefly, three to five millilitres of blood were collected into EDTA tubes from patients with a confirmed diagnosis of uncomplicated *P. falciparum *malaria. The fresh blood samples were then centrifuged to remove the buffy coat and cryopreserved in liquid nitrogen following the method of Rowe *et al *[12] before being transported to Pharmacology and Toxicology Unit, Graduate Programme in Biomedical Sciences, Thammasat University.

### Parasite culture and drug susceptibility assay

Parasites were maintained in *in vitro *culture using the method of Trager and Jensen [13]. *In vitro *susceptibility to chloroquine and a series of related compounds, in the presence or absence of verapamil was tested by monitoring [^3^H]Hypoxanthine uptake [14]. Briefly, each drug was prepared as 10 mM in 50% ethanol for stock solution, and further diluted with RPMI-1640 complete media in desired concentrations. Each well in 96-well plate contained 100 ml of drug solution and 10 ml of 1% parasitaemia and 20% haematocrit. The plates were incubated at 37°C in gas chamber (95% N_2_, 5%O_2_, 5%CO_2_) for 24 hours, then pulsed with 5 ml of [^3^H] hypoxanthine solution (0.1 mCi/ml), and reincubated for an additional 24 hours. The plates were harvested and measured with a 1450 MicroBeta Trilux liquid scintillation and luminescence counter (Wallac Co. Ltd., Finland). The drug concentration that inhibited 50% parasite growth (IC_50_) were determined.

### PCR amplification of *P. falciparum *genes

Genomic DNA was extracted using Chelex-resin (Biorad, USA) according to the method of Wooden *et al *[15]. Genetic variation between parasites was investigated by a multiplex PCR fingerprint technique [15]. Previously published nested and allele-restricted PCR methods were employed to detect *pfmdr1 *N86Y [16] and *pfcrt *mutations [7] respectively. For all assays, the laboratory adapted Thai K1 parasite isolate was used as a positive control for the mutant genotype and the Gambia isolate, G112 (a gift from the Malaria Research Unit, Chulalongkorn University, Thailand) as a positive control for the wild type genotype.

### DNA sequencing analysis

The *pfcrt *gene was amplified from genomic DNA using a nested PCR strategy. Exons 1-2, 3-8 and 9-13 were sequenced independently using the primers shown in Table 1. Each reaction contained 1.5 mM MgCl_2_, 200 mM dNTPs, 1 mM each primer, 1× PCR buffer and 2 U of *Taq *polymerase (Promega, USA) in a total volume of 50 ml. The thermal cycle conditions were 94°C for 3 min, and 35 cycles of 92°C for 30 sec, 55°C for 30 sec, 62°C for 1 min 30 sec and a final extension at 62°C for 5 min. The second round of amplification for exon 1-2 was 94°C for 4 min, 35 cycles of 92°C for1 min, 60°C for 1 min, 72°C for 1 min and a final extension step of 72°C for 5 min. PCR products were purified using Wizard SV gel and PCR clean up system (Promaga, USA) according to the manufacturers instructions. Each amplicon was then cloned with the TOPO TA Cloning kit (Invitrogen Life Technologies) and the positive clones were picked from white or blue colonies following overnight incubation in selection media, S-Gal (Sigma, USA). The plasmid DNA was purified using a MiniPrep Kit (Promega, USA) and digested with the restriction enzyme *EcoRI *to confirm the correct insertion. Finally, sequencing was carried out using the M13 forward and reverse primers and DNA*star *(Lasergene) was utilized for sequence analysis.

**Table 1:** Primers used for *pfcrt *sequencing and real-time PCR quantification

Exon 1-2	
Primary	
E1/2-F	CGACATTCCGATATATTATATTTTTAGAC
E1/2-R	TATATGTGTAATGTTTTATATTGG
Nested	
E1/2-NF	CCGTTAATAATAAATACACGCAG
E1/2-NR	AATGTTTTATATTGGTAGGTGG
Exon 3-8	
Primary	
E3/8-F	CCACCTACCAATATAAAACATTAC
E3/8-R	GTTAAAATATATATAAATGTCTC
Nested	
E3/8-NF	TATATATATATGTATGTATGTTG
E3/8-NR	AATGTCTCTTATAATTTTGAAATT
Exon 9-13	
Primary	
E9/13-F	CTTATAATAAAATTTCAAAATTATAAGAGAC
E9/13-R	GAGATCTCTATACCTTCAACATTATTCC
Nested	
E9/13-NF	GAGACATTTATATATATTTTAAC
E9/13-NR	CCTTATAAAGTGTAATGCG
pfcrt RT-PCR	
pfcrt-F	CTTTATTTGTATGATTATGTTCTTTATTGTTTATTCCTTATTTGGA
pfcrt-R	AACAGGCATCTAACATGGATATAGCAAA
pfcrt-probe	TCGGTGTCGTTCTTTTG
pfmdr RT-PCR	
pfmdr-F	GCATTCGGTTTTTGGTATGGTACAA
pfmdr-R	CCTAATAAAATGGATATAACTGAGGCACCA
pfmdr-probe	ACGAATCAATACCCCAATAAT
EF-1a RT-PCR	
EF-1a-F	GGCAGAAAGAGAAAGAGGTATTACCA
EF-1a-R	CCTGGTGCATCAATGACAGTAAAGA
EF-1a-probe	TTATGGAAATTTGAAACCCC

### *Pfcrt *and *pfmdr1 *real-time reverse transcription PCR quantification

*Pfcrt *and *pfmdr1 *gene expression were determined by real-time reverse transcription PCR. Since *pfcrt *and *pfmdr1 *have previously been reported to be preferentially expressed during the trophozoite state, analysis was restricted to isolates at the trophozoite stage. Total RNA was extracted by standard methodology using Trizol™ (Invitrogen, USA) [17]. For *pfcrt*, EF-1a primers were designed to cross introns. However, this was not possible for *pfmdr1 *since it is a one-exon gene. Therefore, each sample was treated with DNase prior to reverse transcription (Invitrogen, USA) in order to negate the possibility of amplifying genomic DNA. The sequences of forward and reverse primers as well as the sequences of the specific reporter probes (FAM-labelled in all cases) are shown in Table 1. Quantification was achieved by standard *taq*Man methodology as previously reported [18].

#### Detection of gene copy number of pfmdr1 by SYBR Green I real-time PCR

*Pfmdr1 *copy number was determined by SYBR Green I real-time PCR (iCycler, Bio-Rad, USA) using the default thermocycler program for all genes: 10 minutes of pre-incubation at 95°C followed by 40 cycles for 15 seconds at 95°C and one minute at 60°C. The oligonucleotide primers were designed by Ferreira [19] with modification. Individual real-time PCR reaction was carried out in 25 μl volume in a 96-well plate containing 1× buffer (10×), 3.5 mM MgCl_2_, 200 μM dNTPs, 1 mM each of sense and antisense primers, 12.5 ml of Platinum™ PCR SuperMix (Invitrogen, USA). The 2^-ΔΔCt ^method of relative quantification was adapted to estimate copy numbers in *P. falciparum *genes. In this work, genomic DNA extracted from *P. falciparum *3D7, known to harbour a single copy of each gene studied, was used as a calibrator, while Pf-β-actin 1 was served as the house-keeping gene in all experiments. Dd2 genomic DNA was used as a second calibrator containing four copies of *pfmdr1*. A minimum of two experiments was carried out for each gene and each sample. In each experiment, each individual sample was run in duplicate wells and the C_t _of each well was recorded at the end of the reaction.

### Statistical analysis

All data are expressed as mean ± standard deviation. Data were assessed for normality using a Shapiro-Wilk statistical test. A Kruskal-Wallace statistical test was utilized to assess the relationship between mutations in *pfcrt *and *pfmdr1 *and drug IC_50_. Correlations were assessed by Spearman Rank test. Statistical significance for all tests was set at a α = 0.05.

## Results

### Genetic polymorphisms of *P. falciparum *isolates

A total of 95 isolates (excluding the laboratory lines: K1, G112, M12 and T9/94b3) were successfully cultured and analysed. Of these, 13 were obtained from the Tak Province of Thailand, 37 from Kanchanaburi, 16 from Ranong, 1 from Ratchaburi, and 14 were from Chantaburim Provinces. Fourteen isolates were of unknown origin. Tak, Kanchanaburi and Ranong are provinces that lie along the Thai-Myanmar border; the first two areas have been described as 'high mefloquine-resistance areas', whereas the third is described as a 'low mefloquine-resistance area'. Chantaburi Province is located along the Thai-Cambodian border and is defined as a 'high mefloquine-resistance area' (Ministry of Public Health, Thailand [20].

Based on the results of the PCR fingerprints, all but two infections were clonal containing only one parasite population with a single genotype of either *msp1*, *msp2 *or *csp*. Two isolates showed PCR fingerprint suggesting a mixed-infection. Genotyping for *pfcrt *revealed a 100% prevalence of the 76T, 220S, 271E, 326S and 371I mutations (95 of 95 samples) with a 94% prevalence of the 356T Mutation (89 of 95 samples). The K1-type mutation of the *pfmdr1 *was seen in only 5% (5 of 95) of the samples with almost all isolates containing the wild type Asn at position 86.

### *In vitro *drug sensitivity test

A total of 90 isolates were tested for sensitivity to chloroquine (CQ), quinine (QN), mefloquine (MQ), and dihydroartemisinin (DHA). The sensitivity of the isolates to CQ and QN was also assessed in the presence or absence of the resistance reverser verapamil (VP: 5 mM). Sensitivity of the isolates to CQ, QN and MQ was categorized based on IC_50 _values described by Cerutti *et al*, [21].

**Figure 1:**
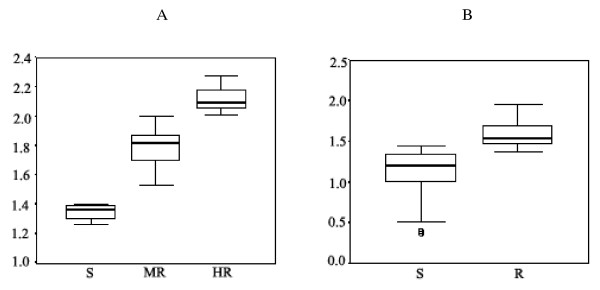
**Box plot between logIC**_**50 **_**and the order of drug susceptibility**. A: CQ was categorised into high resistance (HR: IC_50 _>101 nM), moderate resistance (MR: 30.9 <IC_50 _≤ 100.9 nM) and sensitive (S; IC_50 _≤ 30.9 nM). B: MQ was divided into 2 groups; resistant (R; IC_50 _>24 nM and sensitive (S; IC_50 _≤ 24 nM)

CQ sensitivity was categorized into either highly resistant (HR: IC_50 _>101 nM), moderately resistant (MR: 30.9 <IC_50 _≤ 100.9 nM) or sensitive (S: IC_50 _≤ 30.9 nM) (Figure 1A). Based on these criteria, 30 (32%), 61 (64%), and 4 (4%) isolates were categorized as HR, MR, and S, respectively. The corresponding values for geometric mean (95% CI) IC_50 _were 130.9 (122.1-140.2), 60.8 (56.7-65.3), and 23.0 (20.5-25.5) nM, respectively. All resistant isolates demonstrated a verapamil reversible component of resistance.

QN sensitivity was categorized as either resistant (R: IC_50 _>500 nM) or sensitive (S: IC_50 _≤ 500 nM). Based on these criteria, 1 (1%), and 92 (99%) isolates were categorized as R, and S, respectively. The corresponding values for geometric mean (95% CI) IC_50 _were 654.4 and 144.4 (129.4-161) nM, respectively. All isolates were chemosensitized verapamil.

MQ sensitivity was categorized as either resistant (R: IC_50 _>24 nM) or sensitive (S: IC_50 _≤ 24 nM) (Figure 1B). Based on these criteria, 44 (32%), and 47 (68%) isolates were categorized as R, and S, respectively. The corresponding values for geometric mean (95% CI) IC_50 _were 38.2 (34.4-42.4), and 13.3 (11-16) nM, respectively.

The IC_50 _of DHA for different isolates ranged from 0.37 to 4.87 nM, with the geometric mean (95% CI) of 1.42 (1.24-1.64) nM.

### The relationship between genotype and phenotype

Based on the known mutations in *pfcrt *and *pfmdr1*, there was no statistically significant association between susceptibility of *P. falciparum *isolates to CQ, QN and MQ and *pfcrt *polymorphisms, as well as between susceptibility to CQ and QN and *pfmdr1 *polymorphisms. However, there was a tendency (no statistical significance) of reduced MQ susceptibility in *pfmdr1 *mutant isolates.

### Novel sequence polymorphisms in *pfcrt*

A total of 20 isolates selected from either the moderately or highly CQ resistant subgroups were fully sequenced for the *pfcrt *gene in order to establish if other, as yet unknown mutations could discriminate between these two levels of *in vitro *susceptibility. The derived sequences were compared with the sequences of the 3D7 and Dd2 parasites from the PlasmoDB database. The sequencing results are summarized in Table 2. There were several new mutations identified at a number of novel positions, which conferred an amino acid alteration compared to 3D7. However, it was not possible in this study to directly link these sequence differences with CQ susceptibility. Although most of the parasites had the familiar "CVIET" sequence at positions 72-76, the J9 isolate from Chantaburi contained the novel mutation at codon 76 with a different amino acid substitution K76A. This is a unique position 76 mutation, which has never been reported before in field isolates. In addition, this field isolate carried other mutations at positions S39P and N277D.

**Table 2:** Sequence polymorphism in pfcrt

Isolate/clone	**CQ IC**_**50 **_**(nM)**	Amino acid in *pfcrt *gene										
		
		72	73	74	75	76	198	220	271	326	356	371
3D7	8	C	V	M	N	K	E	A	Q	N	I	R

Dd2	48	C	V	I	E	T	E	S	E	S	T	I

BC1	76	C	V	I	E	T	E	S	E	S	T	I

BC7	54	C	V	I	E	T	E	S	E	S	T	I

BC12	49	C	V	I	E	T	E	S	E	S	T	I

BC22	42	C	V	I	E	T	K*	S	E	S	T	I

BC31	31	C	V	I	E	T	K*	S	E	S	T	I

BC35	57	C	V	I	E	T	K*	S	E	S	T	I

J1	107	C	V	I	E	T	E	S	E	S	T	I

J3	119	C	V	I	E	T	E	S	E	S	T	I

J4	114	C	V	I	E	T	E	S	E	S	T	I

J5	121	C	V	I	E	T	E	S	E	S	T	I

J6	167	C	V	I	E	T	E	S	E	S	T	I

J9	123	C	V	I	E	A**	E	S	E	S	T	I

KS14	67	C	V	I	E	T	E	S	E	S	T	I

KS21	68	C	V	I	E	T	E	S	E	S	T	I

KS28	74	C	V	I	E	T	E	S	E	S	T	I

KS33	55	C	V	I	E	T	E	S	E	S	T	I

KS50	78	C	V	I	E	T	K*	S	E	S	T	I

PCM2	75	C	V	I	E	T	E	S	E	S	T	I

PCM7	51	C	V	I	E	T	E	S	E	S	T	I

RN54	47	C	V	I	E	T	E	S	E	S	T	I

* Glutamic acid = > Lysine

** Lysine == > Alanine

Four of the field isolates categorized as displaying moderate CQ resistance carried a E198K mutation which may be functionally relevant. Furthermore, deletions in the *pfcrt *sequence were observed at position 327 in isolate BC7 and 251 in isolate KS28. These two deletions occurred at exon 2. Interestingly, the A144T or A144F mutation newly described in isolates from the Philippines and Cambodia was not detected in these Thai isolates [8,22].

### *Pfcrt *and *pfmdr1 *gene expression

**Figure 2:**
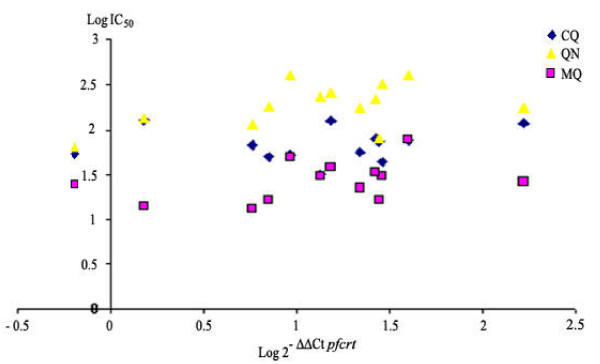
**Correlation analysis between *in vit*ro responses (logIC**_**50**_**) and relative *pfcrt *expression in falciparum samples**. The analysis was deduced using the comparative ΔΔCt method (log 2^-ΔΔCt^) using either EF1-alpha as endogenous control gene.

**Figure 3:**
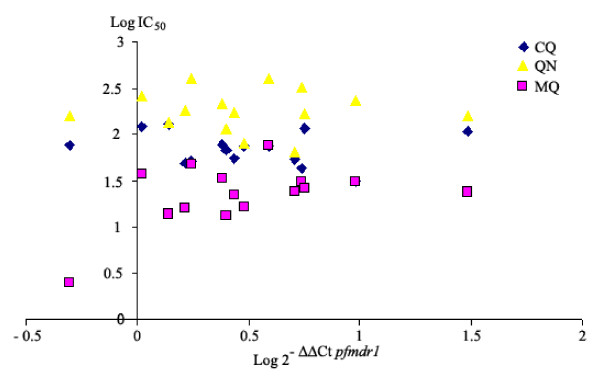
**Correlation analysis between *in vitro *responses (logIC**_**50**_**) and relative *Pfmdr1 *expression in falciparum samples**. The analysis was deduced using the comparative ΔΔCt method (log 2^-ΔΔCt^) using either EF1-alpha as endogenous control gene.

The correlation between *pfcrt *and *pfmdr1 *gene expression (determined by RT-PCR) and *in vitro *response is shown in Figures 2 and 3. In this study, there was no statistically significant correlation between expression levels of either gene and sensitivity to CQ, QN or MQ. Although statistical significance was not achieved, a trend toward higher *pfmdr1 *expression levels in MQ sensitive isolates was observed (*p *= 0.094).

Interestingly, expression of *pfcrt *mRNA could not be detected in two isolates (BC1 and J1) despite confirmation of EF-1a gene expression performed on several occasions. This was not due to any primer mismatch and did not appear to relate to experimental difficulties, although all possibilities cannot be ruled out. This observation needs to be carefully followed up as it has been shown that a *pfcrt *knockout is fatal for the parasite [10].

### *Pfmdr1 *copy number

The *pfmdr1 *copy number was determined by SYBR Green I real-time PCR. The average copy number of Dd2 was 4.4, which was similar to that previously reported [19]. The *pfmdr1 *gene copy number was neither correlated to mRNA expression nor *in vitro *drug susceptibility.

## Discussion

Thailand and its borders have historically been linked with the early emergence of drug resistant parasites. The first report of CQ resistance came from this region in the late 1950's. MQ resistance was reported shortly after its deployment, a situation which was worsened despite doubling the drug dosage for treatment. More recently, the Malaria Control Programme of Thailand has introduced artesunate, an artemisinin derivative, deployed in combination with mefloquine to control these multidrug resistant parasites. With respect to CQ susceptibility, molecular studies have confirmed the role for mutation in the *pfcrt *gene and CQR with a possible modulatory influence coming from the *pfmdr1 *gene. In contrast, MQ susceptibility has been linked to mutation [6,23,24] and gene copy number of *pfmdr1 *[25]. Despite these linkage data, a wide range of observations cannot be fully explained by the currently available genetic evidence. In this study, this issue was investigated in more detail using isolates recently collected from distinct regions of Thailand.

It is unquestionable that genetic polymorphisms of parasite transporter genes have affected the susceptibility to various drugs. In early studies of multidrug resistance in South East Asia, single nucleotide polymorphisms (SNPs) in *pfmdr1 *gene such as N86Y have been identified [26]. In Africa, where the 4-aminoquinoline is the main drug for malaria treatment, the frequency of N86Y polymorphism is high (>50%) [12,16,27-29]. However the frequency of N86Y mutation observed in the present study was relatively low, which is in agreement with its frequency in other South East Asian isolates [3,27,30-33]. This might be due to the influence of MQ pressure since mid 1980s after CQ was dismissed in this region [25,34].

Whilst the association of *pfmdr1 *polymorphisms and drug sensitivity is still a matter of debate, the level of expression of *pfmdr1 *seems to offer a better explanation of anti-malarial drug sensitivity profiles, especially for MQ, QN and artemisinin derivatives. Previous studies have shown the relationship between the increase of *pfmdr1 *gene copy number and multidrug resistance phenotypes including artesunate [35-39]. In one study, over-expression of *pfmdr1 *was found to be correlated with MQ resistance [40]. On the basis that an increase of gene copy number or gene over-expression may be expected to result in a rise in the mRNA, the levels of *pfmdr1 *mRNA and copy number were compared. It is very surprising; the level of mRNA was not correlated to gene copy number. Results failed to reveal an association between *pfmdr1 *expression or gene copy number and drug sensitivity. This is analogous to the reported absence of association between Pgh1 protein expression and CQ sensitivity [41]. The authors note that two isolates (BC1 and J1), which lack *pfcrt *expression have different *pfmdr1 *expression. For the J1 isolate, of which *pfmdr1 *expression was approximately 70-fold over BC1, showed reduced sensitivity to both CQ and MQ. Although the trend of correlation between *pfmdr1 *expression and sensitivity to MQ was observed, due to small number of sample size, a statistically significant correlation could not be achieved. These data suggest that increase of copy number and over-expression of *pfmdr1 *gene may not necessarily confer drug resistance on its own. Nevertheless, the expression and polymorphism of *pfmdr1 *may make a major contribution to drug resistance when present in conjunction with the mutation of other genes [6].

Several studies have described gene mutations in association with CQ-resistance but the exact mechanism of resistance remains unclear. The K76T mutation of *pfcrt *is reported to be highly associated with CQ-resistance. The frequency of this mutation is almost 100% in Thailand. Consequently there seems to be no link between the frequency of this gene mutation and CQ sensitivity. Parasites carrying the K76T mutation with comparable patterns of other *pfcrt *gene mutations still showed a variable degree of CQ-resistance. In fact, the *pfcrt *polymorphisms were similar in all Thai isolates but drug sensitivity profiles were completely different. Data reported from other South East Asian countries, *i.e*., Philippines, showed different mutations carrying K76T and N326D without 220S residue, together with the novel mutations A144T and L160Y [42]. Furthermore, four novel mutations A144F, L148I, I194T and T333S were observed from Cambodian isolates [11]. These mutation patterns were not observed in Thailand. This suggests that CQ-resistance emerged simultaneously but independently in these neighboring endemic regions.

*Pfcrt *polymorphism pattern CVIET was found in all isolates. This pattern is unique to *P. falciparum *isolates from South East Asia. The CVIET pattern, which is different from SVMNT pattern of South America, is more responsive to the calcium channel-blocker verapamil. The chemosensitization phenomenon of verapamil was observed for all isolates even the isolates with lower sensitivity to CQ. Therefore, the CVIET polymorphism pattern seems to confer chemosensitization by verapamil and related compounds.

Full *pfcrt *sequencing revealed the novel 76 haplotype K76A in one isolate. Previously, the substitution of other amino acids at this codon, *i.e*., K76I and/or K76N has been reported only in laboratory strains [7,43]. This allelic type replaces lysine (positively charged amino acid) with alanine (non-charged amino acid), similar to the charge loss found with K76T, K76I and K76N mutations. These data augment the growing body of evidence that substitution of non-charged amino acid appears to result in resistance of the parasite to CQ. In addition, the new mutation at position E198K was detected in 4 out of 20 isolates with high CQ sensitivity (low IC_50_). Based on amino acid sequence, it is speculated that E198K might be located in the fifth of ten PFCRT transmembrane regions, with substitution of glutamic acid (negative charge) to lysine (positive charge). The additional mutation in this position may be critical to the loss of resistance of these parasites to CQ. These findings may support the proposed hypothesis that CQR results from a "charged drug leak", in which the loss of positive charge in the channel of PfCRT might allow the protonated species of CQ to leak out of the digestive vacuole, thus reducing vacuolar CQ concentration and ultimately conferring resistance [44].

Results obtained from transfection studies showed significant correlation between the extent of protein expression and drug sensitivity profile (increased resistance with increased expression) [10]. In contrast, recent observation of *pfcrt *expression and parasites from field isolates showed no correlation between level of mRNA expression and CQ sensitivity [11]. In our study, such absence of correlation between level of mRNA expression and CQ sensitivity was also observed. Furthermore, it was found that no mRNA expression was observed in two isolates. This finding was in contrast with the previous report showing that the knockout of *pfcrt *gene would lead the parasite to death. The undetectable level of mRNA expression could be explained by the low or totally absence of *pfcrt *expression, or the fact that gene expression may not entirely represent protein expression due to post-transcription process.

## Competing interests

The authors declare that they have no competing interests.

## Authors' contributions

WC performed the molecular genetic studies, participated in the sequence alignment, interpretation of data, performed the statistical analysis and drafted the manuscript. SA participated in the design of the study and data analysis. MM participated in sample collection and *in vitro *sensitivity test. DJ, AO and PB participated in the molecular genetic studies and sequence alignment. KN participated in the design of the study and finalized the manuscript. All authors read and approved the final manuscript.
